# Internet use and adolescent development in rural China: A scoping review protocol of research landscape and gaps

**DOI:** 10.1371/journal.pone.0308229

**Published:** 2024-09-11

**Authors:** Linxiao Zhang

**Affiliations:** Factor-Inwentash Faculty of Social Work, University of Toronto, Toronto, Ontario, Canada; University of Botswana, BOTSWANA

## Abstract

**Introduction:**

In the digital era, the Internet has become a necessity in adolescents’ daily lives. Many studies globally are exploring the influences of Internet use on adolescent development, but they focus on the negative impacts of simplistic “screen time” on adolescents’ physical and mental health, rather than both positive and negative influences of multifaceted Internet use on multidimensional adolescent development. Specifically in rural China, adolescents live in disadvantageous and marginalizing contexts, and Internet use is widespread among this population. However, knowledge on Internet use and adolescent development in rural China is fragmented. It is still unclear in what ways Internet use would bring benefits or risks for Chinese rural adolescents’ healthy growth. Therefore, the objective of this scoping review is to identify the current research landscape, gaps, and future directions in rural China contexts. It aims to provide a comprehensive overview of elements, findings, and limitations in existing empirical studies on the influences of Internet use on adolescent development in rural China.

**Materials and methods:**

The standard for conducting this scoping review is the five-stage model proposed by Arksey and O’Malley, and the reporting standard is the Preferred Reporting Items for Systematic Reviews and Meta-Analyses: Extension for Scoping Reviews (PRISMA-ScR). The overall research question is: What are the influences of Internet use on adolescent development in rural China? In consultation with librarians, to locate articles, subject headings (controlled vocabularies) and textwords (keywords) in article titles, abstracts, and author-assigned keywords will be searched in APA PsycInfo (psychology), China National Knowledge Infrastructure (interdisciplinary), Communication Abstracts (media and communication), Education Source (education), MEDLINE (public health), Social Services Abstracts (social work), Social Work Abstracts (social work), and Sociological Abstracts (sociology). The review process via Covidence will consist of two sequential steps based on inclusion/exclusion criteria: the title and abstract review and the full-text review. Then study characteristics and research findings will be charted, and the results will be analyzed and synthesized quantitatively and qualitatively via visualizations and narratives, guided by the typological frameworks of Internet use and adolescent development.

**Discussion:**

The scoping review will be a pioneering review to inform the current research landscape and gaps in the Internet use influences on adolescent development in rural China. It will advance the research agenda on this issue conceptually, theoretically, and empirically. In addition, it can provide contextual implications for designing prevention and intervention programs.

## Introduction

Adolescent Internet use is a social phenomenon and public health consideration worldwide. It neutrally refers to adolescents’ utilization of the Internet and its services, which has both quantitative (time, frequency, etc.) and qualitative (venue, device, platform/app, activity, etc.) attributes [1, 2]. Compared with qualitative Internet use that elaborates on how adolescents use the Internet and what they do online, quantitative Internet use focuses on the amount of Internet use by adolescents.

Many studies in different socio-cultural contexts have inquired into the influences of Internet use on adolescent development, referring to adolescents’ physiological, cognitive, and psychosocial growth [3, 4]. Although with inconsistent findings and even small statistical effects, most of these studies were concerned about negative impacts of general “screen time” or total online hours (quantitative Internet use) on mainly adolescents’ physical and mental health, such as eyesight deterioration, sleep disturbance, obesity, depression, anxiety, and suicidality [5–11]. This scoping review aims to step beyond the globally dominant addiction-based discourse of Internet use and present the diversity of developmental outcomes in rural China. Multifaceted Internet use may have both positive and negative influences on various aspects of adolescent development, including their physical and mental health, intelligence and academics, identity and personality, and socio-emotional skills and behaviors [12–15].

Furthermore, much research in this field does not employ theories and only presents statistical results. Without theoretical dialogues, it is difficult to understand “why” and “how” Internet use affects adolescent development in rural China. In addition, existing studies are scattered among different disciplines, including social work, psychology, sociology, education, public health, and media and communication, which would benefit from systematic combination and integration. The current scoping review aspires to connect empirical evidence with social science theories and bring various disciplines together for a joint discussion on this issue.

Meanwhile, relevant knowledge on Chinese rural adolescents lacks comprehensiveness, and the current scoping review aims to enhance the awareness and contextualization of this issue in rural China. Numerous studies have documented the disadvantageous, lonely, and tedious childhood of both adolescents living with parents and those from left-behind families in rural China. Left-behind adolescents, a considerable group under the background of rapid urbanization and widening urban-rural socio-economic inequality, refer to those whose parent(s) migrate to urban areas for better employment opportunities, and their main caregivers are grandparents. Chinese rural adolescents are growing up with many strains and challenges, such as poverty and economic distress, leisure deprivation, substance misuse, bullying victimization, and domestic abuse and neglect [16–24]. Consequently, adolescent development in rural China falls behind, involving mental unwellness, cognitive deficiencies, and socio-emotional aggression [17–20]. For example, Huang et al. (2017) reported that the prevalence of aggressive behavior among Chinese rural adolescents was 24.3% [20].

Under these marginalizing developmental contexts, Internet use is widespread among Chinese rural adolescents. According to the *2021 National Report on Internet Use by Underage (6–17)* [25], the Internet use prevalence among rural children and adolescents (97.3%) exceeded their urban counterparts (96.7%) for the first time, and their possession and usage rates of smartphones (69.2%; 92.5%) were also higher than the rates among urban groups (58.3%; 90.2%). These urban-rural gaps began to narrow dramatically from 2019/2020 (and finally reversed), driven by e-learning to reduce the urban-rural educational disparity and digital needs during the COVID-19 pandemic. Although the objective digital divide shrinks in terms of popularized Internet infrastructure, access, and hardware in rural China, little attention is paid to adolescents’ soft digital literacy and performance–how they use the Internet with what kinds of developmental outcomes. For example, some studies found that they enjoyed online entertainment, such as video gaming and watching short videos and influencers’ lives [26–28], while long-time Internet use negatively impacted their psychological well-being [29–32].

To conclude, the influence of Internet use on adolescent development is a rising issue in rural China. It is still unclear at the intersection of unique living contexts and transitional puberty period; in what ways would Internet use be an opportunity or challenge for Chinese rural adolescents’ healthy growth? Therefore, we will undertake a scoping review to examine existing studies and provide implications for further research on Internet use and adolescent development in rural China.

## Materials and methods

The conduction of this scoping review will follow the five-stage framework proposed by Arksey and O’Malley [33], which is further improved by Levac et al. [34] and the Joanna Briggs Institute [35]. The result reporting will be guided by the Preferred Reporting Items for Systematic Reviews and Meta-Analysis: Extension for Scoping Reviews (PRISMA-ScR, see S1 Appendix) [36].

### Stage 1: Identify the research question

This scoping review aims to identify the current research landscape, gaps, and future directions in the influences of Internet use on adolescent development in rural China. Research questions include: What aspects of Internet use and adolescent development are included in empirical studies? What positive or negative influences of Internet use on adolescent development have been found? Which theories are used to explain the influences in empirical studies? What research limitations are discussed in current literature?

### Step 2: Identify relevant studies

We will search English journal articles in the following databases in consultation with librarians: Social Work Abstracts (Ovid), APA PsycInfo (Ovid), MEDLINE (Ovid), Social Services Abstracts (ProQuest), Sociological Abstracts (ProQuest), Education Source (EBSCO), and Communication Abstracts (EBSCO). We will search Chinese articles in the monopolistic database, China National Knowledge Infrastructure (CNKI).

Search terms combine four themes: Internet use, adolescent, rural area, and China. Because it is difficult to control the concepts of “influences” and “development” in the search, we will manually screen relevant studies in the third stage without designing them as search terms. Databases will be searched by using subject headings and textwords in titles, abstracts, and author-assigned keywords. An example search string generated via PsycInfo is provided in the S2 Appendix, and the search string will be translated for other databases after the pilot search. The formal search will be performed on June 30, 2024, and search results will be imported into the software, Covidence.

### Stage 3: Study selection

The review process via Covidence will consist of two sequential steps based on inclusion/exclusion criteria: the title and abstract review and then the full-text review. Articles selected in the first step will be reviewed again in the second step. For each step, two research assistants will independently review each article simultaneously, and the lead researcher will resolve their conflicts (determine if an article without consensus between the two reviewers will be included). As a calibration test, the two reviewers will both screen the same sets of 50 abstracts. After reaching a similarity of 90% between the two reviewers [37], the remaining articles will be reviewed independently by themselves.

Inclusion criteria involve: (1) articles published in English or Chinese; (2) full-text peer-reviewed academic journal articles; (3) published between July 1, 2014, and June 30, 2024; (4) empirical studies (quantitative, qualitative, or mixed-methods); (5) research participants: adolescents between 12–17 years old and/or junior/senior high school students (according to China’s educational policy and rural life course); (6) research area: rural China; (7) research topic: the influences of adolescents’ Internet use on their physiological, cognitive, and/or psychosocial development.

Grey literature will be excluded due to the concern about the objective of this scoping review (informing and guiding further scientific research by identifying the present landscape, gaps, and potential directions) and the research quality of studies in grey literature. Articles published before recent ten years will be excluded for the following reasons: the objective of this scoping review, the relative novelty of the issue (especially expanded by the Covid-19 pandemic), and the implications for current policy and practices. Regarding the 5th and 6th criteria, for research with multiple populations and comparative studies, they will be included as long as they contain separate results on adolescents in rural China. Finally, as the scope is to review research on how Internet use affects adolescent development, articles focusing on what influences Internet use (Internet use factors), how to guide or regulate Internet use (Internet use interventions), how Internet use impacts non-developmental areas (such as civic engagement and substance use), or how Internet addiction affects adolescent development will be excluded. “Internet addiction” is a different term from Internet use, and Internet addiction is not included in the research scope. Internet use is understood as neutral and objective behavioral patterns of quantitative and qualitative use (not involving using digital devices unconnected to the Internet); while Internet addiction refers to a disorder–a problematic usage of Internet-based devices and services characterized by being excessive, compulsive, impulsive, and hasty, with associated physical and psychological symptoms [38], though current definitions and conceptualizations of Internet addiction are inconsistent and disputable [39]. Studies that measure Internet use aggregately (such as assessing problematic Internet use via a scale) without capturing the details of quantitative and/or qualitative elements of Internet use will also be excluded. We consider the study selection an iterative process, and will hold team meetings to ensure a consistent understanding of the criteria and modify our review accordingly. The final scoping review will include a PRISMA flow diagram to illustrate the whole screening process.

### Stage 4: Chart the data

We will chart two types of data via Excel: study characteristics and research findings. For study characteristics, one research assistant will record the article title, year, language, study design/methods, location/data level, sample size, and sample demographics (age/educational stage, gender, whether left behind, etc.). For research findings, another research assistant will document aspects of Internet use, aspects of adolescent development, findings related to the influences of Internet use on adolescent development (also positive or negative), mechanism of the influences (if existing mediators, moderators, path analysis, etc.), theories used to explain the influences, and research limitations discussed. The lead researcher will review the charting results. As the third stage, the fourth stage will also be an iterative process. The chart including a full list of selected articles with study characteristics and research findings of each article will be uploaded as an appendix.

### Stage 5: Data summary and synthesis of results

The synthesis will combine visualizations (graphs and tables) and narratives. The results will be analyzed both quantitatively (such as frequencies and proportions of study characteristics) and qualitatively (such as thematic analysis of research findings) based on typological frameworks of Internet use and adolescent development. As mentioned in the introduction, preliminary dimensions of Internet use comprise quantitative use, qualitative use, and quantitative-qualitative use (measurements bridging quantitative and qualitative uses, such as comparing time spent on different Internet devices and online activities). Preliminary dimensions of adolescent development comprise physical and mental health, intelligence and academics, identity and personality, and socio-emotional skills and behaviors [12–15]. Finally, we will discuss implications and directions for future research. To achieve knowledge transfer, the scoping review will be disseminated via the stakeholder seminar, academic conference, and journal article.

## Discussion

This scoping review should be understood in the context of limitations. Firstly, we will not formally assess the research quality of each chosen article, but such an assessment is not a requirement in scoping reviews [40]. Secondly, we will not incorporate grey literature so we may miss some useful sources of research evidence, however, this strategy takes into account our objective and the research quality as mentioned in Stage 3. Thirdly, our search may omit some articles because we employ certain databases with specific strings, while working together with librarians and manually checking reference lists can mitigate this problem.

Although with limitations, the scoping review will be a pioneering review to inform the current research landscape and gaps in the influences of Internet use on adolescent development in rural China. It will advance the research agenda on this issue both conceptually/theoretically and empirically, because the scoping review synthesizes the operationalizations/measurements of Internet use and adolescent development, findings related to the influences in rural China and relevant theories and mechanisms, as well as research limitations discussed in empirical studies. Moreover, it may offer some insights into designing prevention and intervention programs [41]. Existing positive influences can serve as entry points for leveraging Internet use to boost adolescent development in rural China, while negative influences can suggest human service targets in terms of Internet use elements and mechanism factors. By mapping heterogeneity analysis in current studies, we can also identify sub-groups susceptible to positive influences and vulnerable to negative impacts.

## Supporting information

S1 Appendix PRISMA-P (Preferred Reporting Items for Systematic review and Meta-Analysis Protocols) checklist* adapted for the scoping review protocol. (DOC)

S2 Appendix Search string for PsycInfo as a pilot database. (DOCX)
