# Supplementary material for: Internet use and adolescent development in rural China: A scoping review protocol of research landscape and gaps
Source: PLoS One. 2024 Sep 11;19(9):e0308229. doi: 10.1371/journal.pone.0308229 (PMC11389942; doi:10.1371/journal.pone.0308229)
Supplement: S2 Appendix — (DOCX) [file pone.0308229.s002.docx]

**S2 Appendix. Search string for PsycInfo as a pilot database.**

1. Internet Usage/

2. (Internet or online or cyber or web or virtual or digital or remote or screen* or "information and communication technolog*" or ICT or social media or social networking site* or SNS or video gam* or e-learning or e-education or short video* or video clip* or live stream* or smartphone* or smart phone* or mobile phone* or cell phone* or cellphone* or smart device* or mobile device* or computer* or laptop* or app*).tw.

3. 1 or 2

4. exp Adolescent Development/

5. (adolescen* or teen* or youth* or juvenile* or middle school* or secondary school* or high school*).tw.

6. 4 or 5

7. exp Rural Environments/

8. (rural or village* or countryside* or left-behind).tw.

9. 7 or 8

10. Chinese Cultural Groups/

11. (China or Chinese).tw.

12. 10 or 11

13. 3 and 6 and 9 and 12
